# Genetic Analysis of a Horizontal Resistance Locus *BLMR2* in *Brassica napus*

**DOI:** 10.3389/fpls.2021.663868

**Published:** 2021-05-25

**Authors:** Qiang Zhang, Hanna Dandena, Madison McCausland, Huizhi Liu, Zheng Liu, Wen Xu, Genyi Li

**Affiliations:** Department of Plant Science, University of Manitoba, Winnipeg, MB, Canada

**Keywords:** *Brassica napus*, *Leptosphaeria maculans*, *BLMR2*, cytochrome p450, fine mapping

## Abstract

*Leptosphaeria maculans* causes blackleg disease in *Brassica napus*. The blackleg disease is mainly controlled by resistance genes in *B. napus*. Previous studies have shown that the blackleg resistant *BLMR2* locus that conferred horizontal resistance under field conditions, is located on chromosome A10 of *B. napus*. The purpose of this study is to fine map this locus and hence identify a candidate gene underlying horizontal resistance. The spectrum of resistance to *L. maculans* isolates of the resistance locus *BLMR2* was analyzed using near isogenic lines, resistant, and susceptible cultivars. The results showed that this locus was horizontally resistant to all isolates tested. Sequence characterized amplified regions (SCAR), simple sequence repeats (SSR), and single nucleotide polymorphism (SNP) markers were developed in the chromosome region of *BLMR2* and a fine genetic map was constructed. Two molecular markers narrowed *BLMR2* in a 53.37 kb region where six genes were annotated. Among the six annotated genes, *BnaA10g11280D/BnaA10g11290D* encoding a cytochrome P450 protein were predicted as the candidate of *BLMR2*. Based on the profiling of pathogen induced transcriptome, three expressed genes in the six annotated genes were identified while only cytochrome *P450* showed upregulation. The candidate corresponds to the gene involved in the indole glucosinolate biosynthesis pathway and plant basal defense in *Arabidopsis thaliana*. The molecular markers identified in this study will allow the quick incorporation of the *BLMR2* allele in rapeseed cultivars to enhance blackleg resistance.

## Introduction

*Brassica napus* (oilseed rape/canola) is an important crop used for edible oil production worldwide. Blackleg, caused by *Leptosphaeria maculans*, is one of the most devastating diseases in *B. napus* (canola, oilseed, and rapeseed) production. There are both qualitative and quantitative types of resistance to fungal pathogens like *L. maculans* (Rouxel et al., 2003; Raman et al., 2012, 2013). Qualitative resistance is race-specific and depends on the presence of a single resistance (R) gene in plant and corresponding avirulence (Avr) genes in pathogen (Ansan-Melayah et al., 1998). In contrast, quantitative resistance or horizontal resistance is race non-specific, which may be mediated by several genes and expressed from the seedling to adult plant stages, conferring only partial resistance to all races of the pathogen (Delourme et al., 2006; Rimmer, 2006). Identification and incorporation of resistance genes in *Brassica* species to produce resistant cultivars is an efficient approach to combat blackleg disease in *B. napus* (Hayward et al., 2012).

To date, over a dozen loci including *Rlm1-10*, *LepR1* to *LepR4*, *BLMR1*, and *BLMR2* conferring resistance to *L. maculans* have been mapped in the cultivated *Brassica* species (Yu et al., 2005, 2008, 2013; Delourme et al., 2006; Rimmer, 2006; Long et al., 2011; Larkan et al., 2013). However, the effectiveness of these resistance genes is decreased after they are used in production for a few years, and the genes need to be replaced with other novel genes (Li et al., 2003; Rouxel et al., 2003; Kutcher et al., 2007). For example, *LepR3* in Surpass 400 that provides race-specific resistance to the fungal pathogen *L. maculans* suffered a major defeat in Australia in 2004 (Li et al., 2003; Sprague et al., 2006). On the other hand, studies on rice (Maeda et al., 2006) and barley (Van Berloo et al., 2001) have confirmed that quantitative genes conferred effective resistance. Quantitative resistance likely remains effective over time than qualitative resistance (Kaur et al., 2009; Brun et al., 2010; Zhang et al., 2016). Therefore, it is important and necessary to identify and combine qualitative and quantitative genes for durable blackleg resistance.

It is very difficult to identify the genes for quantitative trait loci in the complex genome of *B. napus* because little information is available on the genetic control of quantitative resistance to *L. maculans*. In previous study, the blackleg quantitative resistance gene *BLMR2* identified in Surpass 400 showed horizontal resistance under field conditions (Dandena et al., 2019). A few blackleg resistance genes including *Rlm2, LepR3, BLMR2*, and *LepR2* have been mapped on *B. napus* chromosome A10. Among these the highly resistant *Rlm2* and *LepR3* are allelic and function as receptors (Larkan et al., 2013, 2014, 2015). Characterization of the *BLMR2* and *LepR2* need further studies although these genes are likely different from the other R genes based on the map position and phenotype (Long et al., 2011; Larkan et al., 2016). *BLMR2* segregated as a single dominant allele and has a distinctly intermediate phenotype when cotyledon inoculated with the *L. maculans* isolate 87-41 (Long et al., 2011). This allowed the development of NILs (W + BLMR2) using molecular marker assisted backcrossing along with progeny testing of recombinants.

In this report, *BLMR2* was fine mapped in *B. napus* with the use of sequence characterized amplified regions (SCAR) and simple sequence repeats (SSR) markers. Furthermore, inoculation assay with individual isolates showed that *BLMR2* has race non-specific resistance to all the isolates tested. These results lay a foundation for utilizing the blackleg resistance *BLMR2* allele in developing resistant *B. napus* cultivars that effectively control *L. maculans*.

## Materials and Methods

### Materials for Testing Horizontal Resistance

Three near isogenic lines containing resistance *BLMR1*, *BLMR2*, and *Rlm2* alleles were developed using the two resistant cultivars Surpass 400 and Glacier, and a susceptible cultivar Westar. *BLMR1* and *BLMR2* were derived from the cross between Surpass 400 and Westar (Long et al., 2011) while *Rlm2* was derived from the cross between Glacier and Westar. *BLMR1* and *BLMR2* were separated at BC1F3 based on differential phenotypic interaction with the *L. maculans* isolate 87-41 and molecular marker data (Long et al., 2011). From crosses of Surpass 400 × Westar and Glacier × Westar, F3 and F1 progeny, respectively, were backcrossed to Westar four to five times and molecular marker assisted selection (MAS) was implemented during backcrossing (Dandena et al., 2019). Individuals containing homozygous *BLMR1, BLMR2*, or *Rlm2* alleles were selected based on molecular markers and inoculation assays to obtain three isogenic lines in Westar background, named as W + BLMR1 (BC_4_F_4_), W + BLMR2 (BC_4_F_4_), and W + Rlm2 (BC_4_F_2_), respectively. The near isogenic lines, together with two resistant cultivars Glacier and Quinta, and the susceptible Westar were used to perform interaction analysis.

### Mapping Populations

The BC_1_F_3_ plants carrying *BLMR2/blmr2 alleles* from the cross of *B. napus* cultivar Surpass 400 and Westar were backcrossed to Westar *(blmr2/blmr2)* to produce BC_2_, BC_3_, and BC_4_ populations, and the BC_4_ was selfed to obtain BC_4_F_4_. All plants used in backcrossing and selfing were phenotyped through inoculation and genotyped using the flanking molecular markers. The BC_4_ F_3_ and BC_4_F_4_ were inoculated with *L. maculans* isolate 87-41 to test cotyledon resistance. Segregation ratios of resistant to susceptible individuals in the F_4_ and BC_4_ were analyzed with χ2 test of goodness of fit. A total of 5,952 BC_3_ individuals were used to fine map the resistance locus.

#### Preparation of *L. maculans* Inoculum

To test the horizontal and race-non-specific resistance of *BLMR2*, 24 *L. maculans* isolates were used in cotyledon assay. These pathogen isolates were selected from the collection at the University of Manitoba. The inoculum of all isolates was prepared, and cotyledons were wounded and inoculated as described previously (Long et al., 2011). Disease reactions were rated in 12–16 days after inoculation according to the classification of 0–9 (Chen and Fernando, 2006).

### DNA Extraction and Development of SSR and SCAR

A modified CTAB extraction procedure as described by Li and Quiros (2001) was used to extract DNA. The *Brassica rapa* genomic sequence^1^ was used to identify SSR loci and primers covering SSR were designed to amplify the specific loci in *B. napus.* SCAR loci were developed using a similar procedure as SSR markers except sequencing the targets from Surpass 400 and Westar. The A genome-specific primers were used to amplify Surpass 400 and Westar to identify insertions/deletions of the targets which were used to develop SCAR markers.

### Detection of SCAR and SSR

A five fluorescent dye color set 6-FAM, VIC, NED, PET and LIZ was used for signal detection with an ABI 3100xl Genetic Analyzer (Thermo Fisher Scientific, Toronto, ON, Canada). The LIZ color was the standard and other four were used to label primers of SSR and SCAR. For SSR and SCAR detection, the genome-specific primers were used to obtain PCR products containing SSR or deletion/insertion positions. A 10 μl PCR mixture contained two genome-specific primers and one labeled M13 primer, 50 ng of genomic DNA, 0.375 mM dNTP, 1X PCR buffer, 1.5 mM MgCl_2_ and 1 U of Taq polymerase. The PCR running program was 94°C, 3 min; 94°C, 1 min; 58°C with −0.8°C each cycle 1 min and 72°C, 1 min for 5 cycles; 94°C, 1 min; 57°C, 1 min and 72°C, 1 min for 25 cycles. The PCR products were separated in the ABI 3100 Genetic analyzer. The data were collected and analyzed with ABI GenScan software and further transferred into images for scoring using Genographer software.

### Sequencing of the Candidate Gene

Fully expanded cotyledons were inoculated using *L. maculans* isolate 87-41 (isolate-inoculated) or water (mock-inoculation). Four days after inoculation, cotyledon samples from eight individual plants per sample were pooled and ground in liquid nitrogen. Total RNA was extracted using Trizol reagent (Thermo Fisher Scientific, Toronto, ON, Canada). RNA quality was determined using 1% agarose gel and Nanodrop. cDNA was synthesized using total RNA and the SuperScript^TM^ III kit following manufacturer’s protocol (Thermo Fisher Scientific, Toronto, ON, Canada). Then, primers (Table 1) were used to amply the full-length cDNA of the candidate gene of *BLMR2* and cloned into the TA cloning vector with the TOPO^®^ TA Cloning^®^ Kit (Thermo Fisher Scientific, Toronto, ON, Canada). Positive clones were selected to extract plasmid DNA using the standard mini preparation protocol. The plasmid DNA was sequenced using the BigDye^TM^ terminator v3.1 cycle sequencing kit (Thermo Fisher Scientific, Toronto, ON, Canada). Full length cDNA sequence was assembled using SeqMan software.

**TABLE 1:** Primers for all applications.

Primer names	Sequence (5′-3′)	Applications
N10-56	TAATACTGGTTAATTATGCT	Mapping
RN10-56	ACAGTACATTCACGTTCTAG	Mapping
N10-47	ACTGGCCTATGGATGACGTT	Mapping
RN10-47	AATCCAGCAGTAGACCCCAT	Mapping
N10BA	CGAAAGTAAGAAGAGCAAGA	Mapping
N10BB	GATACTCTAGTTGTTGACAA	Mapping
N10-45	CAGAAGAAGAAGGATATGGT	Mapping
RN10-45	TCCAGTTAACCAATGCTGGT	Mapping
N10-43	CTAAGAAATTTCCTATGACAC	Mapping
RN10-43	TTGTCAATGTCTCATGCTAA	Mapping
N10-39	GGCTGCGTTGTTTCATACCT	Mapping
N10-39	ATGTGGGAGCTGAGGTTGTC	Mapping
RN10-39	GTCCTTAGTTGGTCCACTGT	Mapping
N10-34	CGAGCAGCAAATCCATATCC	Mapping
RN10-34	CAATTTTGTATTTTCTTATGGAAACTG	Mapping
N10-38	TTCAACATTTCTCCGCGATA	Mapping
RN10-38	TTTCCATCTGCTTCCACCTAA	Mapping
N10-37	CAGTCCTGACTTTGCCATCA	Mapping
N10-37	ACAGGCGAGAGGTTTGAAGA	Mapping
N10-40	CACAATTTCTGGTATACAGATTG	Mapping
RN10-40	CTTTGGAGCGAATTGTTGAAG	Mapping
10BM1	TGCAGGCAATTATTTCAGTGG	MAS
10BM2	AGCTTATGTTAGGTGGAAG	MAS
HN62F	ATGGATTACATTTTGCTCTTATTG	TA cloning
HN65R	TTAAGCCAAAAGATTAGTCATA	TA cloning
ACTIN-1F	CGATGGTGAGGACATTCAGC	RT-PCR
ACTIN-1R	AGAGAGAAAGAACAGCCTGGAT	RT-PCR
MM1F	ACAAGTAGACCAACCCAA	RT-PCR
MM1R	CCACAAACTCGCCATCGC	RT-PCR

### Quantitative Analysis of Candidate Gene Expression

Total RNA was extracted from cotyledon samples of NIL, W + BLMR2 and Westar inoculated with *L. maculans* isolate 87-41 at 4 days after inoculation (dai). cDNA was synthesized using the SuperScript IV First-Strand Synthesis kit (Thermo Fisher Scientific, Toronto, ON, Canada). Quantitative real-time PCR (qRT-PCR) was conducted using SYBR Green Master Mix (Bio-Rad) according to manufacturer’s recommendation (Bio-Rad). The P450 gene specific primers were used to amplify the gene (Table 1). The actin gene (BnaA01g19850D) was used as a control for normalization. Relative gene expression was calculated by the 2^–ΔΔCT^ method.

## Results

### Resistance to *L. maculans* Isolates

Three near isogenic lines W + BLMR1, W + BLMR2, and W + Rlm2, along with Wester, Glacier, and Quinta were inoculated with 24 isolates. The results showed that only W + BLMR2 was resistant to all tested isolates, indicating that the resistance of *BLMR2* was horizontal and race non-specific. *BLMR1* and *Rlm2* showed differential interactions with the tested isolates and the two resistant cultivars Glacier and Quinta also showed different resistant spectra while Westar was susceptible to all isolates as expected (Table 2).

**TABLE 2:** Testing of resistance spectra of three near isogenic lines and three cultivars in *Brassica napus* using 24 isolates of Leptosphaeria maculans*.

Isolates	W + BLMR2	W + BLMR1	W + Rlm2	Glacier	Quinta	Westar
87-1	R	S	S	S	S	S
M4-1	R	S	S	S	S	S
M4-2	R	S	S	S	S	S
10Aridries-dk	R	S	S	S	R	S
3-12-01	R	S	R	S	R	S
08-01-05	R	S	R	S	R	S
9STONEWALL	R	S	R	S	R	S
3-15-03	R	S	R	R	R	S
6NBW-01	R	S	R	R	R	S
89-3	R	S	R	R	R	S
ND04-05-01	R	S	R	R	R	S
86-12	R	S	R	R	R	S
4-09-109	R	R	S	R	S	S
7-02-01	R	R	S	R	S	S
53-31	R	R	S	R	S	S
Lifolle	R	R	S	R	S	S
PL03-02-01	R	R	S	R	S	S
PL03-42-06	R	R	S	R	S	S
3-54-01	R	R	S	R	R	S
4-09-01	R	R	S	R	R	S
3-01-Roland	R	R	S	R	R	S
4-09-107	R	R	R	R	R	S
87-41	R	R	R	R	R	S
Lifolle 5	R	R	R	S	R	S

*All isolates from the collection of University of Manitoba. Three near isogenic lines W + BLMR1, W + BLMR2, W + Rlm2, two resistant cultivars Glacier and Quinta, and susceptible cultivar Westar; R representing 0–4 ratings, S representing 5–9 ratings.

### Segregation of BLMR2 in the Mapping Populations

**FIGURE 1:**
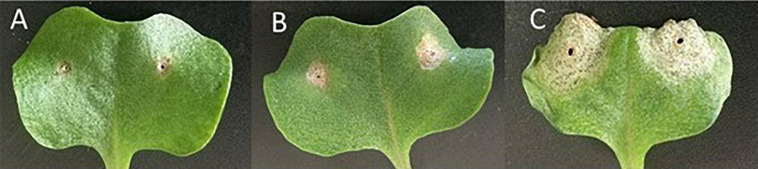
Cotyledons of Surpass 400, near isogenic line W + BLMR2 and Westar, 14 days after inoculation (dai) of the pycnidiospore suspension (2 × 10^7^ spores/mL) of *Leptosphaeria maculans* isolate 87-41. Surpass 400 showing high resistance **(A)**; W + BLMR2 showing intermedium resistance **(B)**; and Westar with the susceptible phenotype **(C)**.

In the mapping populations of *BLMR2*, 656 F_4_ individuals were used to confirm the segregation ratio of *BLMR2*. There were 513 resistant plants and 143 susceptible plants showing a 3:1 segregation ratio (χ^2^ test, *p* > 0.05). In the BC_4_ mapping population of 831 individuals, there were 438 resistant plants and 393 susceptible plants showing a 1:1 segregation ratio (χ^2^ test, *p* > 0.05), suggesting that one dominant resistance allele is responsible for the *BLMR2* resistance to blackleg (Figure 1).

### Fine Mapping of the Resistance Gene

**FIGURE 2:**
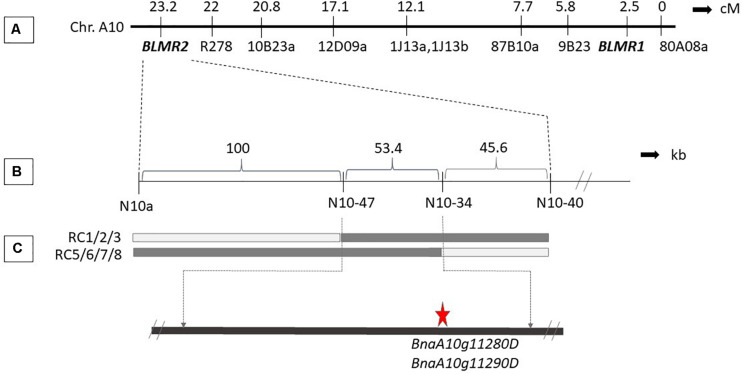
Genetic and physical map of *BLMR2* locus on Chromosome A10 of *Brassica napus*. Genetic map showing *BLMR1* and *BLMR2* (Long et al., 2011; **A**). The flanking SSR and SCAR markers used in the fine mapping of *BLMR2* **(B)**. Molecular marker data of the recombinants with resistant phenotype showing overlapping *BLMR2* region corresponding to 57.3 kb physical interval. The candidate *BLMR2* gene is indicated **(C)**.

Sequence characterized amplified region (SCAR) and SSR markers were used to map *BLMR2* on chromosome A10. Using 1,632 BC_4_ plants, six recombinant individuals were obtained. Their selfed progeny was inoculated with *L. maculans* isolate 87-41 to confirm recombinants. Then, more sequence-based SCAR and SSR were developed to fine map the *BLMR2* locus. Another 4,320 BC_4_ individuals were inoculated with *L. maculans* isolate 87-41 to identify another 14 recombinants. Using all 20 recombinants, *BLMR2* was narrowed in a small region between molecular markers N10-47 and N10-43 after their phenotypes and genotypes were analyzed (Table 3 and Figure 2). Selfed progeny of all recombinants was also tested to confirm the recombination in the BC_4_F_4_. The chromosome region of molecular markers N10-47 and N10-43 was located from 9494993 to 9548367 in the reference genome sequence of chromosome A10^2^, spanning a 53.37 kb region. In the reference sequence, seven genes are annotated while two genes *BnaA10g11280D* and *BnaA10g11290D* were re-annotated into one gene based on the full length of cDNA.

**TABLE 3:** Phenotypesand genotypes of 20 recombinants in the BC_3_ population of Brassica napus*.

Recombinant	Phenotype	N10a	N10-56	N10-47	N10b	N10-45	N10-43	N10-39	N10-34	N10-37	N10-38	N10-40
RC01	R	A	A	AB	AB	AB	AB	AB	AB	AB	AB	AB
RC02	R	A	A	AB	AB	AB	AB	AB	AB	AB	AB	AB
RC03	R	A	A	AB	AB	AB	AB	AB	AB	AB	AB	AB
RC04	R	AB	AB	AB	AB	AB	AB	AB	AB	AB	AB	AB
RC05	R	AB	AB	AB	AB	AB	AB	AB	AB	A	A	A
RC06	R	AB	AB	AB	AB	AB	AB	AB	AB	A	A	A
RC07	R	AB	AB	AB	AB	AB	AB	AB	AB	A	A	A
RC08	R	AB	AB	AB	AB	AB	AB	AB	AB	A	A	A
RC09	S	A	A	A	A	A	AB	AB	AB	AB	AB	AB
RC10	S	A	A	A	A	A	A	AB	AB	AB	AB	AB
RC11	S	A	A	A	A	A	A	A	AB	AB	AB	AB
RC12	S	A	A	A	A	A	A	A	AB	AB	AB	AB
RC13	S	A	A	A	A	A	A	A	AB	AB	AB	AB
RC14	S	A	A	A	A	A	A	A	A	AB	AB	AB
RC15	S	A	A	A	A	A	A	A	A	AB	AB	AB
RC16	S	A	A	A	A	A	A	A	A	AB	AB	AB
RC17	S	A	A	A	A	A	A	A	A	AB	AB	AB
RC18	S	A	A	A	A	A	A	A	A	A	AB	AB
RC19	S	AB	AB	AB	A	A	A	A	A	A	A	A
RC20	S	AB	AB	AB	A	A	A	A	A	A	A	A

*R, resistance; S, susceptible phenotype; A, genotype of Westar; AB, heterozygous genotypes (B, genotype of *BLMR2* in Surpass 400).

Full length cDNA of the candidate gene was sequenced from both the near isogenic line W + BLMR2 with *BLMR2* in Westar background and Westar. Comparison of the cDNA sequences showed that there are six polymorphisms in W + BLMR2 compared to the background genotype Westar while only one of these six nucleotide changes results in one difference of amino acid (AA) (Supplementary Data 1). RNA-seq data in the previous report (NCBI Archive under the BioProject accession number PRJNA378851, Zhou et al., 2019) were analyzed to compare the expression of the six genes in the fine mapped region of *BLMR2* and the results showed that three of the six genes were expressed while only the candidate gene was upregulated after inoculation (Supplementary Data 2).

### Analysis of Expression of the Candidate Gene by qPCR

**FIGURE 3:**
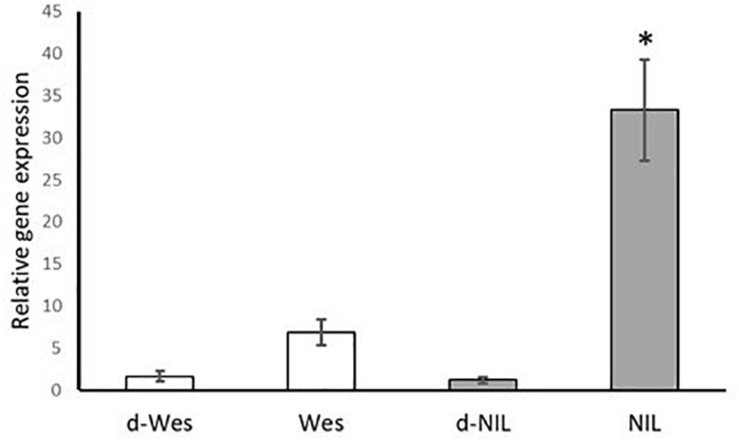
Validation of the expression of *Brassica napus CYP81F2* gene in the resistant near isogenic line (NIL) W + BLMR2 and susceptible Westar using qRT-PCR at 4 days after inoculation (dai) with *Leptosphaeria maculans* isolate 87-41. Samples d-Wes is water check of Westar; Wes, inoculated Westar; d-NIL, water check of NIL; NIL, inoculated NIL. The relative gene expression value is normalized using the Actin gene (*BnaA01g19850D*). Error bar shows standard deviation of the mean based on three biological replicates. The asterisk (^∗^) represents significant difference from all the other samples based on Tukey test, *p* < 0.05.

In addition, qRT-PCR was used to determine the relative gene expression of the candidate gene in the resistant W + BLMR2 and Westar. The relative fold change in the accumulation of *B. napus CYP450* transcript was significantly higher in the pathogen (*L. maculans* isolate 87-41) inoculated resistant W + BLMR2 at 4 dai compared to pathogen inoculated Westar or mock checks (Figure 3). Our results suggest that *CYP450* possibly plays a role in *L. maculans* induced defense response in *B. napus*.

## Discussion

It is hypothesized that quantitative resistance genes work by a complex interaction of many response genes to pathogens, and any individual gene does not show strong effect, therefore there is less selective pressure on the fungus (Zhu et al., 1993; Delourme et al., 2008). Mapping and eventually cloning these genes will facilitate the transfer and pyramiding of multiple resistance genes with different resistance spectra through molecular marker-assisted selection in *B. napus* (Long et al., 2011). In this paper, the disease reactions of *BLMR2* to a range of isolates of *L. maculans* were analyzed. The results showed that *BLMR2* is a horizontal resistance locus to all the isolates of the blackleg pathogen, compared to typical R genes and resistant cultivars. In the previous study, the results in field testing showed that *BLMR2* conferred resistance to blackleg under field conditions, suggesting that this resistance gene is very useful to the breeding of resistant cultivars in *B. napus* (Dandena et al., 2019).

In this study, *BLMR2* was fine mapped in a 53.37 kb region where six genes were annotated. Of these six genes, one was identified as the candidate gene of *BLMR2* based on the analysis of gene expression. The candidate is homologous to *CYP81F2* in *Arabidopsis thaliana.* Several studies showed that *CYP81F2* catalyzes the modification of indole glucosinolate (IGS) which involves the accumulation of defensive secondary metabolites (Abdel-farid et al., 2010; Wiesner et al., 2013). In Arabidopsis, its hydrolytic products produced by myrosinases (*PEN2* and *PEN3*) are involved in innate immune response to pathogens (Bednarek et al., 2009; Clay et al., 2009). The *CYP81F2* and *PEN2* dependent hydrolytic products are also associated with callose deposition in FLG22-triggered basal immunity (Bednarek et al., 2009; Clay et al., 2009). While it remains elusive whether genes involving the glucosinolate biosynthesis are linked to defense response induced by *L. maculans* in *B. napus*, a complex pattern of IGS accumulation in *B. rapa – L. maculans* interaction was observed (Abdel-farid et al., 2010). Furthermore, Robin et al. (2017) associated upregulation of *CYP81F2 (Bol026044)* gene in the moderate blackleg resistant cabbage cultivar with increased IGS accumulation at the seedling stage. Recent global transcriptomic studies identified multiple genes involved in the IGS biosynthesis in *B. napus – L. maculans* incompatible interaction (Becker et al., 2017; Zhou et al., 2019). However, the mechanisms underlying horizontal resistance are rarely reported, and hence the findings in this study suggest that secondary metabolic pathways such as the biosynthetic pathways of glucosinolates may play a role in plant resistance to various diseases. However, how the change of AA in the DNA sequence confers the horizontal resistance in this study need to be investigated in the future.

Plant disease resistance is classified in many ways, vertical vs horizontal, qualitative vs quantitative, race-specific vs race-non-specific, a high vs an intermediate level of resistance. Typical R genes such as NBS-LRR, receptor like proteins (RLP) and receptor like kinases (RLK) have been cloned since their phenotypes can be easily observed (Dangl and Jones, 2001). For some diseases such as sclerotinia diseases in rapeseed, sunflower and soybean and fusarium head blight in cereal crops, no vertical, qualitative or race-specific resistance has been identified so no typical R genes for these diseases has been cloned (Behla et al., 2017; Mesterhazy, 2020). In this study, the horizontal resistance of *BLMR2* has been stressed since it conferred intermediate resistance under field conditions (Dandena et al., 2019) and showed intermediate resistance to all isolated tested under controlled environmental conditions. Several characteristics of the resistance of the *BLMR2* locus need to be addressed. First, its resistance is race-non-specific and intermediate while the resistance is dominant and resistant in all heterozygous genotypes tested. Second, single allele of this locus confers relatively strong resistance, so the phenotypes of this locus were relatively easy to be scored (Figure 1). The accurate scores of phenotypes made it possible to perform fine mapping and narrow the gene into a small chromosome region. Third, this kind of resistance is assumed to be controlled by polygenes and very difficult to be transferred from cultivar to cultivar while the *BLMR2* locus can be easily transferred using molecular markers targeting the six mutations in the DNA sequence (Supplementary Data 1). Finally, unlike all previous reports where horizontal resistance is hypothesized to have a minor effect of one locus in resistance controlled by multiple loci (Tian et al., 2006; Skowrońska et al., 2020), the resistance locus *BLMR2* confers a relative strong effect so the near isogenic lines showed the level of resistance which can meet the standard of blackleg resistance in Canada field trials (Canola Council of Canada).

## Conclusion

The availability of accurate phenotypes and a large population aided in the precise mapping of the *BLMR2* locus. With a combination of *BLMR2* fine-mapping, molecular marker assisted development of NILs and comparative physical mapping, the candidate gene for *BLMR2* as a homolog of *CYP81F2 (At5g57220)* in Arabidopsis was identified. Markers identified in this study can be used to transfer this horizontal resistance from cultivar to cultivar using MAS or gene pyramiding for resistance durability.

## Data Availability Statement

The datasets presented in this study can be found in online repositories. The names of the repository/repositories and accession number(s) can be found below: https://www.ncbi.nlm.nih.gov/, PRJNA378851.

## Author Contributions

QZ, HD, and GL designed the experiment and wrote the manuscript. QZ and HD finished the most experiments. MM, HL, ZL, and WX collected the data of some experiments. All authors contributed to the article and approved the submitted version.

## Conflict of Interest

The authors declare that the research was conducted in the absence of any commercial or financial relationships that could be construed as a potential conflict of interest.

## Footnotes

1 http://brassicadb.cn

2 www.genoscope.cns.fr
