# Supplementary material for: Genetic Analysis of a Horizontal Resistance Locus BLMR2 in Brassica napus
Source: Front Plant Sci. 2021 May 25;12:663868. doi: 10.3389/fpls.2021.663868 (PMC8186441; doi:10.3389/fpls.2021.663868)
Supplement: Supplementary file 1 [file Data_Sheet_1.PDF]

Supplementary Material

Supplementary Data S1. cDNA sequences of the *Brassica napus CYP81F2* homolog in Westar and W+BLMR2. The coding sequences are composed of 576 bases – exon1; 288 bases – exon2(green); 612 bases – exon3; sequence variations were observed in exons 1 and 3 (red). **(A)** and translated protein alignment shows one amino acid difference between Westar and W+BLMR2 **(B).**

(A) cDNA alignment

>Westar

ATGGATTACATTTTGCTCTTATTGCCACTCGTATTGTTTCTACTAGCTTACAAATTCTTAT
TCTCATCTAAGAGTTTCAATCTTCCACCAGGACCAACTCCCTTTCCCATCGTCGGCAACC
TCCACCTCGTGAAACCACCGGTGCACCGTCTCTTCCGTCGTTTCGCGGACAAGTACGGTG
ACATCTTCTCCCTCCGTTACGGCTCTCGCCAAGTCGTCGTGATCTCTTCCTTGCCCCTCGT
CAGAGAATGCTTTACTGGTCAGAACGACGTTATTTTAACGAACCGACCGCATTTTCTGAC
CGCAAAGTACGTTGCTTACGACTACACCACGGTTGGAACCGCCGCATATGGCGACCACT
GGCGTAATCTCCGCCGTATTTGCTCTCTTGAGATCCTTTCCTCTAACCGTCTCACTGGATT
CCTCTCCGTTCGTAAAGACGAGATCCGACGGTTGCTCACGAAACTCTCACGTGACTATA
ATGGCCAAGTCGTTGAGCTTGAGCCTCTTCTTGCAGATTTGACGTTCAATAATATTGTCC
GTATGGTCACTGGGAGACGTTACTACGGAGACCAGGTTCACAACAAGGAAGAAGCGAA
CCTATTCAAGAAGCTAGTGACGCAGATCAACGACAATAGTGGTGCGAGCCATCCAGGAG
ATTATTTACCAATTCTCAAAGTTTTCGGACACGGCTACGAGAAGAAAGTGAAAGCACTC
GGCGAAGCCATGGACACTTTCTTGCAGCGACTGCTCGACGATTGCCGTAGAGATGGAGA
GAGCAACACAATGCTTAGTCATCTGTTGTCTTTACAAGTAGACCAACCCAAGTATTACA
GTGACGTCATCATCAAAGGCCTCATGCTCAGTATGATGCTTGCGGGGACGGATACTGCA
GCCGTGACACTAGAATGGGCGATGGCGAGTTTGTTGAAAAGTCCTGAAGTGTTGAAGAA
GGCGAAAGCCGAGATAGATGATAAGATTGGACATGAACGTTTGGTCGACGAACCGGAC
ATTTTGAATCTCCCTTATCTCCAAAACATAGTTTCTGAGACCTTCCGACTGTGTCCAGCC
GCACCACTCCTTGTACCACGTTCTCCTTCTGAAGACCTCAAGATTGGCGGATACGACATA
CCGCGTGGCACCATCGTACTAGTGAATTCTTGGGCCATCCATAGAGATCCAAGGCTTTG
GGATGAGCCTGAGAGGTTCATGCCAGAGCGGTTTGAGGACAAAGAAGCTGCCAATAAT
AATAAGCTTATGATGTTTGGGAACGGACGAAGGACGTGTCCCGGTGCGGCTTTGGGTCA
AAGGATGGTGTCGTTGGCTTTAGGATCGTTGATTCAATGCTTTGACTGGGAAAAAGTCA
ACGGTGAGGAAATTGATATGACCGAAAATCCTGGAATGGCTATGCGCAAGCTCGTGCCG
TTACGAGCCGTTTGCCATCAGCGTCCCATTATGACTAATCTTTTGGCTTAA

>W+BLMR2

ATGGATTACATTTTGCTCTTATTGCCACTCGTATTGTTTCTACTAGCTTACAAATTCTTAT
TCTCATCTAAGAGTTTCAATCTTCCACCAGGACCAACTCCCTTTCCCATCGTCGGCAACC
TCCACCTCGTGAAACCACCGGTGCACCGTCTCTTCCGTCGTTTCGCGGAGAAGTACGGTG
ACATCTTCTCCCTCCGTTACGGCTCTCGCCAAGTCGTCGTGATCTCTTCCTTGCCCCTCGT
CAGAGAATGCTTTACTGGTCAGAACGACGTTATTTTAACGAACCGACCGCATTTTCTGAC

CGCAAAGTACGTTGCTTACGACTACACCACGGTTGGAACCGCCGCATATGGCGACCACT
GGCGTAATCTCCGCCGTATTTGCTCTCTTGAGATCCTTTCCTCTAACCGTCTCACTGGATT
CCTCTCCGTTCGTAAAGACGAGATCCGACGGTTGCTCACGAAACTCTCACGTGACTATA
ATGGCCAAGTCGTTGAGCTTGAGCCTCTTCTTGCAGATTTGACGTTCAATAATATTGTCC
GTATGGTCACTGGGAGACGTTACTACGGAGACCAGGTTCACAACAAGGAAGAAGCGAA
CCTATTCAAGAAGCTAGTGACGCAGATCAACGACAATAGTGGTGCGAGCCATCCAGGAG
ATTATTTACCAATTCTCAAAGTTTTCGGACACGGCTACGAGAAGAAAGTGAAAGCACTC
GGCGAAGCCATGGACACTTTCTTGCAGCGACTGCTCGACGATTGCCGTAGAGATGGAGA
GAGCAACACAATGCTTAGTCATCTGTTGTCTTTACAAGTAGACCAACCCAAGTATTACA
GTGACGTCATCATCAAAGGCCTCATGCTCAGTATGATGCTTGCGGGGACGGATACTGCA
GCCGTGACACTAGAATGGGCGATGGCGAGTTTGTTGAAAAGTCCTGAAGTGTTGAAGAA
GGCGAAAGCCGAGATAGATGATAAGATTGGACATGAACGTTTGGTCGACGAACCAGAC
ATTTTGAATCTCCCTTATCTCCAAAACATAGTTTCCGAGACCTTCCGACTGTGTCCAGCC
GCACCACTCCTTGTCCCACGTTCTCCTTCTGAAGACCTCAAGATTGGCGGATACGACATA
CCGCGTGGCACCATCGTACTAGTGAATTCTTGGGCCATCCATAGAGATCCAAGGCTTTG
GGATGAGCCTGAGAGGTTCATGCCAGAGCGGTTTGAGGACAAAGAAGCTGCCAATAAT
AATAAGCTTATGATGTTTGGGAACGGACGAAGGACGTGTCCCGGTGCGGCTTTGGGTCA
GAGGATGGTGTCGTTGGCTTTAGGATCGTTGATTCAATGCTTTGACTGGGAAAAAGTCA
ACGGTGAGGAAATTGATATGACCGAAAATCCTGGAATGGCTATGCGTAAGCTCGTGCCG
TTACGAGCCGTTTGCCATCAGCGTCCCATTATGACTAATCTTTTGGCTTAA

```
Westar          ATGGATTACATTTTGCTCTTATTGCCACTCGTATTGTTTCTACTAGCTTACAAATTCTTA
W+BLMR2         ATGGATTACATTTTGCTCTTATTGCCACTCGTATTGTTTCTACTAGCTTACAAATTCTTA
                ************************************************************

Westar          TTCTCATCTAAGAGTTTCAATCTTCCACCAGGACCAACTCCCTTTCCCATCGTCGGCAAC
W+BLMR2         TTCTCATCTAAGAGTTTCAATCTTCCACCAGGACCAACTCCCTTTCCCATCGTCGGCAAC
                ************************************************************

Westar          CTCCACCTCGTGAAACCACCGGTGCACCGTCTCTTCCGTCGTTTCGCGGACAAGTACGGT
W+BLMR2         CTCCACCTCGTGAAACCACCGGTGCACCGTCTCTTCCGTCGTTTCGCGGAGAAGTACGGT
                ************************************************** *********

Westar          GACATCTTCTCCCTCCGTTACGGCTCTCGCCAAGTCGTCGTGATCTCTTCCTTGCCCCTC
W+BLMR2         GACATCTTCTCCCTCCGTTACGGCTCTCGCCAAGTCGTCGTGATCTCTTCCTTGCCCCTC
                ************************************************************

Westar          GTCAGAGAATGCTTTACTGGTCAGAACGACGTTATTTTAACGAACCGACCGCATTTTCTG
W+BLMR2         GTCAGAGAATGCTTTACTGGTCAGAACGACGTTATTTTAACGAACCGACCGCATTTTCTG
                ************************************************************

Westar          ACCGCAAAGTACGTTGCTTACGACTACACCACGGTTGGAACCGCCGCATATGGCGACCAC
W+BLMR2         ACCGCAAAGTACGTTGCTTACGACTACACCACGGTTGGAACCGCCGCATATGGCGACCAC
                ************************************************************

Westar          TGGCGTAATCTCCGCCGTATTTGCTCTCTTGAGATCCTTTCCTCTAACCGTCTCACTGGA
W+BLMR2         TGGCGTAATCTCCGCCGTATTTGCTCTCTTGAGATCCTTTCCTCTAACCGTCTCACTGGA
                ************************************************************

Westar          TTCCTCTCCGTTCGTAAAGACGAGATCCGACGGTTGCTCACGAAACTCTCACGTGACTAT
W+BLMR2         TTCCTCTCCGTTCGTAAAGACGAGATCCGACGGTTGCTCACGAAACTCTCACGTGACTAT
```

```
                    ************************************************************

Westar              AATGGCCAAGTCGTTGAGCTTGAGCCTCTTCTTGCAGATTTGACGTTCAATAATATTGTC
W+BLMR2             AATGGCCAAGTCGTTGAGCTTGAGCCTCTTCTTGCAGATTTGACGTTCAATAATATTGTC
                    ************************************************************

Westar              CGTATGGTCACTGGGAGACGTTACTACGGAGACCAGGTTCACAACAAGGAAGAAGCGAAC
W+BLMR2             CGTATGGTCACTGGGAGACGTTACTACGGAGACCAGGTTCACAACAAGGAAGAAGCGAAC
                    ************************************************************

Westar              CTATTCAAGAAGCTAGTGACGCAGATCAACGACAATAGTGGTGCGAGCCATCCAGGAGAT
W+BLMR2             CTATTCAAGAAGCTAGTGACGCAGATCAACGACAATAGTGGTGCGAGCCATCCAGGAGAT
                    ************************************************************

Westar              TATTTACCAATTCTCAAAGTTTTCGGACACGGCTACGAGAAGAAAGTGAAAGCACTCGGC
W+BLMR2             TATTTACCAATTCTCAAAGTTTTCGGACACGGCTACGAGAAGAAAGTGAAAGCACTCGGC
                    ************************************************************

Westar              GAAGCCATGGACACTTTCTTGCAGCGACTGCTCGACGATTGCCGTAGAGATGGAGAGAGC
W+BLMR2             GAAGCCATGGACACTTTCTTGCAGCGACTGCTCGACGATTGCCGTAGAGATGGAGAGAGC
                    ************************************************************

Westar              AACACAATGCTTAGTCATCTGTTGTCTTTACAAGTAGACCAACCCAAGTATTACAGTGAC
W+BLMR2             AACACAATGCTTAGTCATCTGTTGTCTTTACAAGTAGACCAACCCAAGTATTACAGTGAC
                    ************************************************************

Westar              GTCATCATCAAAGGCCTCATGCTCAGTATGATGCTTGCGGGGACGGATACTGCAGCCGTG
W+BLMR2             GTCATCATCAAAGGCCTCATGCTCAGTATGATGCTTGCGGGGACGGATACTGCAGCCGTG
                    ************************************************************

Westar              ACACTAGAATGGGCGATGGCGAGTTTGTTGAAAAGTCCTGAAGTGTTGAAGAAGGCGAAA
W+BLMR2             ACACTAGAATGGGCGATGGCGAGTTTGTTGAAAAGTCCTGAAGTGTTGAAGAAGGCGAAA
                    ************************************************************

Westar              GCCGAGATAGATGATAAGATTGGACATGAACGTTTGGTCGACGAACCGGACATTTTGAAT
W+BLMR2             GCCGAGATAGATGATAAGATTGGACATGAACGTTTGGTCGACGAACCAGACATTTTGAAT
                    ***********************************************.************

Westar              CTCCCTTATCTCCAAAACATAGTTTCTGAGACCTTCCGACTGTGTCCAGCCGCACCACTC
W+BLMR2             CTCCCTTATCTCCAAAACATAGTTTCCGAGACCTTCCGACTGTGTCCAGCCGCACCACTC
                    ************************** *********************************

Westar              CTTGTACCACGTTCTCCTTCTGAAGACCTCAAGATTGGCGGATACGACATACCGCGTGGC
W+BLMR2             CTTGTCCCACGTTCTCCTTCTGAAGACCTCAAGATTGGCGGATACGACATACCGCGTGGC
                    *****.******************************************************

Westar              ACCATCGTACTAGTGAATTCTTGGGCCATCCATAGAGATCCAAGGCTTTGGGATGAGCCT
W+BLMR2             ACCATCGTACTAGTGAATTCTTGGGCCATCCATAGAGATCCAAGGCTTTGGGATGAGCCT
                    ************************************************************

Westar              GAGAGGTTCATGCCAGAGCGGTTTGAGGACAAAGAAGCTGCCAATAATAATAAGCTTATG
W+BLMR2             GAGAGGTTCATGCCAGAGCGGTTTGAGGACAAAGAAGCTGCCAATAATAATAAGCTTATG
                    ************************************************************

Westar              ATGTTTGGGAACGGACGAAGGACGTGTCCCGGTGCGGCTTTGGGTCAAAGGATGGTGTCG
W+BLMR2             ATGTTTGGGAACGGACGAAGGACGTGTCCCGGTGCGGCTTTGGGTCAGAGGATGGTGTCG
                    ***********************************************.************

Westar              TTGGCTTTAGGATCGTTGATTCAATGCTTTGACTGGGAAAAAGTCAACGGTGAGGAAATT
W+BLMR2             TTGGCTTTAGGATCGTTGATTCAATGCTTTGACTGGGAAAAAGTCAACGGTGAGGAAATT
                    ************************************************************

Westar              GATATGACCGAAAATCCTGGAATGGCTATGCGCAAGCTCGTGCCGTTACGAGCCGTTTGC
W+BLMR2             GATATGACCGAAAATCCTGGAATGGCTATGCGTAAGCTCGTGCCGTTACGAGCCGTTTGC
                    ******************************* ****************************
```

```
Westar          CATCAGCGTCCCATTATGACTAATCTTTTGGCTTAA
W+BLMR2         CATCAGCGTCCCATTATGACTAATCTTTTGGCTTAA
                ************************************
```

(b) Protein sequence alignment

>Westar

MDYILLLLPLVLFLLAYKFLFSSKSFNLPPGPTPFPIVGNLHLVKPPVHRLFRRFADKYGDIFS
LRYGSRQVVVISSLPLVRECFTGQNDVILTNRPHFLTAKYVAYDYTTVGTAAYGDHWRNLR
RICSLEILSSNRLTGFLSVRKDEIRRLLTKLSRDYNGQVVELEPLLADLTFNNIVRMVTGRRY
YGDQVHNKEEANLFKKLVTQINDNSGASHPGDYLPILKVFGHGYEKKVKALGEAMDTFLQ
RLLDDCRRDGESNTMLSHLLSLQVDQPKYYSDVIIKGLMLSMMLAGTDTAAVTLEWAMAS
LLKSPEVLKKAKAEIDDKIGHERLVDEPDILNLPYLQNIVSETFRLCPAAPLLVPRSPSEDLKI
GGYDIPRGTIVLVNSWAIHRDPRLWDEPERFMPERFEDKEAANNNKLMMFGNGRRTCPGA
ALGQRMVSLALGSLIQCFDWEKVNGEEIDMTENPGMAMRKLVPLRAVCHQRPIMTNLLA

>W+BLMR2

MDYILLLLPLVLFLLAYKFLFSSKSFNLPPGPTPFPIVGNLHLVKPPVHRLFRRFAEKYGDIFSL
RYGSRQVVVISSLPLVRECFTGQNDVILTNRPHFLTAKYVAYDYTTVGTAAYGDHWRNLRR
ICSLEILSSNRLTGFLSVRKDEIRRLLTKLSRDYNGQVVELEPLLADLTFNNIVRMVTGRRYY
GDQVHNKEEANLFKKLVTQINDNSGASHPGDYLPILKVFGHGYEKKVKALGEAMDTFLQR
LLDDCRRDGESNTMLSHLLSLQVDQPKYYSDVIIKGLMLSMMLAGTDTAAVTLEWAMASL
LKSPEVLKKAKAEIDDKIGHERLVDEPDILNLPYLQNIVSETFRLCPAAPLLVPRSPSEDLKIG
GYDIPRGTIVLVNSWAIHRDPRLWDEPERFMPERFEDKEAANNNKLMMFGNGRRTCPGAAL
GQRMVSLALGSLIQCFDWEKVNGEEIDMTENPGMAMRKLVPLRAVCHQRPIMTNLLA

```
Westar          MDYILLLLPLVLFLLAYKFLFSSKSFNLPPGPTPFPIVGNLHLVKPPVHRLFRRFADKYG

W+BLMR2         MDYILLLLPLVLFLLAYKFLFSSKSFNLPPGPTPFPIVGNLHLVKPPVHRLFRRFAEKYG

                ********************************************************:***


Westar          DIFSLRYGSRQVVVISSLPLVRECFTGQNDVILTNRPHFLTAKYVAYDYTTVGTAAYGDH

W+BLMR2         DIFSLRYGSRQVVVISSLPLVRECFTGQNDVILTNRPHFLTAKYVAYDYTTVGTAAYGDH

                ************************************************************


Westar          WRNLRRICSLEILSSNRLTGFLSVRKDEIRRLLTKLSRDYNGQVVELEPLLADLTFNNIV

W+BLMR2         WRNLRRICSLEILSSNRLTGFLSVRKDEIRRLLTKLSRDYNGQVVELEPLLADLTFNNIV

                ************************************************************
```

```
Westar          RMVTGRRYYGDQVHNKEEANLFKKLVTQINDNSGASHPGDYLPILKVFGHGYEKKVKALG
W+BLMR2         RMVTGRRYYGDQVHNKEEANLFKKLVTQINDNSGASHPGDYLPILKVFGHGYEKKVKALG
                ************************************************************

Westar          EAMDTFLQRLLDDCRRDGESNTMLSHLLSLQVDQPKYYSDVIIKGLMLSMMLAGTDTAAV
W+BLMR2         EAMDTFLQRLLDDCRRDGESNTMLSHLLSLQVDQPKYYSDVIIKGLMLSMMLAGTDTAAV
                ************************************************************

Westar          TLEWAMASLLKSPEVLKKAKAEIDDKIGHERLVDEPDILNLPYLQNIVSETFRLCPAAPL
W+BLMR2         TLEWAMASLLKSPEVLKKAKAEIDDKIGHERLVDEPDILNLPYLQNIVSETFRLCPAAPL
                ************************************************************

Westar          LVPRSPSEDLKIGGYDIPRGTIVLVNSWAIHRDPRLWDEPERFMPERFEDKEAANNNKLM
W+BLMR2         LVPRSPSEDLKIGGYDIPRGTIVLVNSWAIHRDPRLWDEPERFMPERFEDKEAANNNKLM
                ************************************************************

Westar          MFGNGRRTCPGAALGQRMVSLALGSLIQCFDWEKVNGEEIDMTENPGMAMRKLVPLRAVC
W+BLMR2         MFGNGRRTCPGAALGQRMVSLALGSLIQCFDWEKVNGEEIDMTENPGMAMRKLVPLRAVC
                ************************************************************

Westar          HQRPIMTNLLA
W+BLMR2         HQRPIMTNLLA
                ***********
```

Supplementary Data S2. Data showing the log2FC values of genes in the fine-mapped region of the inoculated W+BLMR2 and Westar against their mock controls

Gene_ID	Arabidopsis gene homolog	Gene Annotations	Differential Expression	
			W+BLMR2	Westar
BnaA10g11240D	AT5G57200	Putative clathrin assembly protein At5g57200	0.00	0.00
BnaA10g11250D	-	-	0.00	0.00
BnaA10g11260D	AT5G57210	Ypt/Rab-GAP domain of gyp1p superfamily protein	0.20	-0.58

BnaA10g11270D	-	-	0.00	0.00
BnaA10g11280D	AT5G57220	cytochrome P450, family 81, subfamily F, polypeptide 2	5.62	2.21
BnaA10g11290D	AT5G57220	cytochrome P450, family 81, subfamily F, polypeptide 2	3.78	1.74
BnaA10g11300D	AT5G57230	Thioredoxin superfamily protein	-0.59	0.15
